# Interplay between Müller cells and microglia aggravates retinal inflammatory response in experimental glaucoma

**DOI:** 10.1186/s12974-021-02366-x

**Published:** 2021-12-24

**Authors:** Xin Hu, Guo-Li Zhao, Meng-Xi Xu, Han Zhou, Fang Li, Yanying Miao, Bo Lei, Xiong-Li Yang, Zhongfeng Wang

**Affiliations:** 1 State Key Laboratory of Medical Neurobiology and MOE Frontiers Center for Brain Science, Institutes of Brain Science, Fudan University, Shanghai, 200032 China; 2 Institute of Neuroscience and Third Affiliated Hospital, Henan Provincial People’s Hospital, Henan Eye Institute, Henan Eye Hospital, People’s Hospital of Zhengzhou University, Zhengzhou University, Zhengzhou, 450003 China

**Keywords:** Glaucoma, Müller cells, Microglia, Activation, Inflammatory response, ATP/P2X7 receptor

## Abstract

**Background:**

Glaucoma, the leading cause of irreversible blindness, is a retinal neurodegenerative disease, which results from progressive apoptotic death of retinal ganglion cells (RGCs). Although the mechanisms underlying RGC apoptosis in glaucoma are extremely complicated, an abnormal cross-talk between retinal glial cells and RGCs is generally thought to be involved. However, how interaction of Müller cells and microglia, two types of glial cells, contributes to RGC injury is largely unknown.

**Methods:**

A mouse chronic ocular hypertension (COH) experimental glaucoma model was produced. Western blotting, immunofluorescence, quantitative real-time polymerase chain reaction (q-PCR), transwell co-culture of glial cells, flow cytometry assay, ELISA, Ca^2+^ image, and terminal deoxynucleotidyl transferase dUTP nick end labeling (TUNEL) techniques were employed to investigate the interaction of Müller cells and microglia, and its underlying mechanisms in COH retina.

**Results:**

We first showed that Müller cell activation in mice with COH induced microglia activation through the ATP/P2X7 receptor pathway. The activation of microglia resulted in a significant increase in mRNA and protein levels of pro-inflammatory factors, such as tumor necrosis factor-α and interleukin-6. These inflammatory factors in turn caused the up-regulation of mRNA expression of pro-inflammatory factors in Müller cells through a positive feedback manner.

**Conclusions:**

These findings provide robust evidence, for the first time, that retinal inflammatory response may be aggravated by an interplay between activated two types of glial cells. These results also suggest that to reduce the interplay between Müller cells and microglia could be a potential effective strategy for preventing the loss of RGCs in glaucoma.

**Supplementary Information:**

The online version contains supplementary material available at 10.1186/s12974-021-02366-x.

## Background

Glaucoma, the leading cause of irreversible blindness, is characterized by optic nerve degeneration, which results from the apoptotic death of retinal ganglion cells (RGCs). The mechanisms underlying RGC apoptosis in glaucoma are extremely complicated, but an abnormal cross-talk between retinal glial cells and RGCs is generally thought to be involved [1, 2]. In glaucoma, Müller cells and microglia, two major types of retinal glial cells, are activated and the activation of glial cells is commonly characterized by the so-called “Janus-faced” nature. That is, it may have a beneficial effect, neuroprotective for normal functioning of neurons by releasing neurotrophic factors and antioxidants, and a deleterious effect on retinal neurons by releasing a variety of cytotoxic factors, such as tumor necrosis factor-α (TNF-α), interleukins (IL), and nitric oxide (NO) [2–8].

In the glaucomatous retina, activated Müller cells are characterized by upregulated expression of glial cytoskeletal proteins, such as glial fibrillary acidic protein (GFAP) and vimentin. In the activated state, microglia display enhanced proliferation, migration from the region largely restricted to the inner retina to the ganglion cell layer (GCL), morphological change from a ramified to an amoeboid shape [4], in addition to increased expression of translocator protein (TSPO), a biomarker of microglia activation [9]. While there is evidence suggesting that the cross-talk between Müller cells and microglia may be involved in retinal diseases [10, 11], little is known about how these two cell populations interact in glaucoma to play a role in RGC dysfunction. It is quite evident that an exploration of the mechanisms underlying the interaction of glial cells may be essential not only for understanding the pathogenesis of glaucoma, but also for developing new treatment strategy of glaucoma. In this study, we investigated whether and how Müller cells and microglia interact with each other in mice with chronic ocular hypertension (COH). Our results show that Müller cell activation induces the release of ATP in these mice, thus causing the activation of microglia through the ATP/P2X7 receptor (P2X7R) pathway. Activated microglia in turn enhance retinal inflammatory response caused by activation of Müller cells, thereby aggravate RGC loss in glaucoma. We also show that activated microglia affect the functional conditions of Müller cells in a non-gliosis manner. These data suggest a possibility that appropriate reduction of the interplay between Müller cells and microglia could be an effective way for preventing the loss of RGCs in glaucoma.

## Methods

### Animals and mouse COH model

All experiments were conducted in accordance with the National Institutes of Health guidelines for the Care and Use of Laboratory Animals, and approved by the animal care committee of Institutes of Brain Science at Fudan University. C57BL/6J and MacGreen mice were purchased from SLAC Laboratory Animal Co., Ltd. (Shanghai, China) and The Jackson Laboratory (Hancock County, USA), respectively. *P2X7R*^*−/−*^ mice were a generous gift from Dr. Yuqiu Zhang at Fudan University. All mice were housed under a constant temperature and 12-h light/dark schedule, with normal food and water ad libitum. In this study, total 820 mice were used, among which 320 were for Western blot experiments, 168 for immunohistochemistry, 240 for primary glial cell cultures, 20 for Ca^2+^ images, 48 for flow cytometry assay, and 24 for terminal dUTP nick end labeling (TUNEL) staining.

Mouse COH model was produced by injection of the micro-magnetic beads (2 μL, diameter ≈ 10 μm, BioMag^®^ Superparamagnetic Iron Oxide, Bangs Laboratories, Ins, USA) into the anterior chamber of the right eyes, referring to the procedure previously described in detail [12, 13]. In brief, the mice were anesthetized by 2% pentobarbital sodium (40 mg/kg, i.p.) and the surgical eyes were further anesthetized by 0.4% oxybuprocaine hydrochloride eyedrop (Benoxil, Santen Pharmaceutical Co. Ltd, Osaka, Japan). Tropicamide eye drops (5 mg/mL) were used to dilate the pupils. Micro-magnetic beads (diameter ≈ 10 μm, BioMag^®^ Superparamagnetic Iron Oxide, Bangs Laboratories, Ins) (2 μL) were slowly injected into the anterior chamber under the aid of an OPMI VISU 140 microscope (Carl Zeiss, Jena, Germany). After the injection, the beads were evenly distributed around the iridocorneal angle using a handheld magnet (0.45 Tesla). Sham-operated treatment was performed on the eyes of other mice, following a similar procedure except for injecting the same volume of normal saline. Chlortetracycline ointment was smeared on surface of the eye ball to prevent infection after operation. The measurement of intraocular pressure (IOP) was conducted in the morning to minimize the circadian deviation, using a handheld digital rebound tonometer (TonoLab, Icare, Finland). The IOPs of both eyes were recorded before operation (baseline), immediately after operation (G0d), and on consecutive days after operation (G1d, G2d, and so on), and weekly thereafter (G1w, G2w, and so on). Successful COH model was defined as the IOP elevation being > 5 mmHg relative to the control eye for at least 7 consecutive days [12]. Once the IOP elevation declined to a value of less than 5 mmHg as compared with that of control eye after a single injection of the magnetic microspheres, the animal was discarded and excluded for further study.

### Primary retinal microglia and Müller cell cultures

The culture procedures for retinal microglia and Müller cells basically followed those reports previously [14] with minor modifications. In briefly, retinas isolated from newborn (5-day-old) mice were cut into pieces and digested with 0.25% trypsin (#25200072, Gibco, Life Technologies, Rockville, MD, USA) for 3 × 5 min at 37 °C. After centrifuging, the dissociated cells were re-suspended in the Dulbecco’s modified Eagle medium (DMEM/F12; #11330057, Gibco), supplemented with 10% fetal bovine serum (FBS; #16140071, Gibco), 100 U/mL penicillin and 100 μg/mL streptomycin (#15140122, Gibco), and then cultured in 75-cm^2^ flasks pre-coated with poly-d-lysine (#7280, Sigma-Aldrich, St. Louis, MO, USA). Microglia purification was conducted when mixed retinal cells were cultured for 12–14 days. Microglia on the top of the cell layer were detached by forcibly flapping the flasks. The detached cells, which were composed of 95% microglia, were seeded into 24-well plates or transwell inserts (#3470, Corning, USA). Müller cell purification was conducted using the remaining cells in flasks [14] and Müller cells of the third-generation were used for experiments. Goat anti-Iba1 (1:1000, #019-19741, FUJIFILM Wako Pure Chemical Corporation, Osaka, Japan) and rabbit anti-GS (glutamine synthase; 1:2000, #MAB302, Millipore, Billerica, MA, USA) antibodies were used to identify the purity of microglia and Müller cells, respectively. The purity of cultured microglia and Müller cells was 92.7 ± 0.02% and 91.8 ± 0.01%, respectively. The experiments were performed at least in triplicate.

### Transwell co-culture of retinal Müller cells and microglia

To analyze how activated Müller cells affected retinal microglia, purified retinal microglia and Müller cells were, respectively, seeded onto 24-well plate and onto the Transwell permeable support membrane inserts (0.4 μm, Corning, USA). After 24 h growing separately, Müller cells were activated by 100 μM 3,5-dihydroxyphenylglycine (DHPG) treatment for 6 h. Microglia were then co-cultured with (1) empty inserts; (2) inserts containing normal Müller cells; (3) inserts containing activated Müller cells; (4) inserts containing activated Müller cells while microglia were pretreated with 10 μM DHPG for 2 h; (5) inserts containing Müller cells pretreated with the connexin-43 (Cx43) specific blocker Gap26 (200 μM) or 2 h followed by 100 μM DHPG treatment for 6 h. To analyze the influence of activated microglia on Müller cells, the protocol for cells seeding was similar as mentioned above. Microglia were activated by 100 μM 2′(3′)-O-(4-benzoylbenzoyl) adenosine 5′-triphosphate (BzATP) treatment for 6 or 12 h. Müller cells were co-cultured with (1) empty inserts; (2) inserts containing normal microglia; (3) inserts containing activated microglia; (4) inserts containing activated microglia in the presence of the TNF-α receptor 1 (TNFR1) inhibitor R7050 (10 μM). After co-culture was finished, the exposed Müller cells or microglia were washed with PBS and harvested for further mRNA or protein analysis and immunofluorescent staining.

### Western blotting

Western blot analysis was conducted as previously described [15–18]. Briefly, the retinas were homogenized. The concentration of total proteins in cell lysate supernatant were detected by standard bicinchoninic acid assay kit (#23227, Thermo Scientific Pierce, USA). The extracted whole protein samples (1.0 μg/μL, 10 μL) were resolved by 8–12% SDS-PAGE gels accordance to the protein molecular weight, and then electroblotted onto PVDF membranes (#03010040001, Roche, Switzerland) using a Mini-PROTEAN 3 Electrophoresis System and Mini Trans-Blot Electrophoretic Transfer System (Bio-Rad, Hercules, CA, USA). The membranes were then incubated overnight at 4 °C with the following primary antibodies: rabbit anti-GAPDH (1:10,000, #2118, Cell Signaling Technology, Danvers, MA, USA), mouse anti-Actin (1:10,000, #A5316, Sigma-Aldrich, USA), rabbit anti-TSPO (1:1000, #ab109497, Abcam, Cambridge, MA, USA), rabbit anti-P2X7R (1:1000, #APR-004, Alomone Labs, Israel), rabbit anti-P2X4R (1:1000, #APR-024, Alomone Labs, Israel), mouse anti-GFAP (1:1000, #G3893, Sigma-Aldrich, USA), mouse anti-CaMKII (phospho T286) (1:1000, #ab171209, Abcam), rabbit anti-CaMKIIα (1:1000, #ab92332, Abcam), which was followed by being incubated with corresponding horseradish-peroxidase-conjugated secondary antibodies (1:10,000, #715-035-020 for donkey anti-mouse and #711-035-152 for donkey anti-rabbit, Jackson ImmunoResearch Labs, Wes Grove, PA, USA), and then incubated with enhanced chemifluorescent reagent (#QI219296, Thermo Scientific Pierce^®^, USA). The blots were imaged with a digital imager (FluorChem E System, ProteinSimple, USA) and protein bands were quantitatively analyzed with Alpha View software (Cell Biosciences, Inc.). In this study, both GADPH and β-actin were used as the internal reference, which was chose depending on the molecular weights of target proteins. All experiments were performed at least in triplicate.

### Immunofluorescent staining

Immunofluorescence staining was performed following the procedure described in detail previously [18–20]. In short, for retinal tissues, eyecups of the anesthetized mice were quickly isolated without perfusion fixation, and then fixed with 4% paraformaldehyde (PFA) in 0.01 M phosphate buffer saline (PBS, pH 7.4) for 2 h at 4 °C. After dehydration with graded sucrose solutions (2 h in 10%, 2 h in 20% and overnight in 30%) at 4 °C, the eyecups were embedded in OCT compound (Tissue Tek, Torrance, CA, USA) and the retinas were vertically sectioned at a thickness of 14 µm on a freezing microtome (Leica, Nussloch, Germany). Cultured glial cells grown on round coverslips were fixed with 4% PFA for 20 min. The retinal slices and the cultured glial cells were blocked for 2 h at room temperature in solution containing 5% bovine serum albumin (BSA) and 0.1% Triton X-100 in PBS, and they were incubated with the following primary antibodies at 4 °C for 48 h: goat-anti-Iba1 (1:500, #ab5076, Abcam), rabbit anti-GS (glutamine synthase; 1:2000, #MAB302, Millipore, Billerica, MA, USA), rabbit anti-TSPO (1:500, #ab109497, Abcam), mouse anti-NFAT1 (1:200, #ab2772, Abcam), rabbit anti-NFkB p65 (1:500, #sc-372, Santa Cruz Biotechnology, Santa Cruz, CA, USA). Immunoreactive proteins were visualized by incubating with corresponding cy3- or 488-conjugated secondary antibodies (1:500, #711-165-152 for Cy^TM^3-conjugated donkey anti-rabbit, #715–545-150 for Alexa Fluor 488-conjugated donkey anti-mouse, #705-545-147 for Alexa Fluor 488-conjugated donkey anti-goat) (Jackson ImmunoResearch Labs). After washing, the coverslips or sections were mounted with anti-fade mounting medium (#SLBW7464, Sigma-Aldrich, USA) and DAPI staining (#10236276001, Roche, Switzerland). Images were photographed with an Olympus FV1000 confocal laser-scanning microscope.

### Detection of RGC apoptosis

TUNEL assay was performed on whole flat-mounted retinas [17]. The DeadEnd Fluorometric TUNEL System G3250 kit was used to detect RGC apoptosis according to the manufacturer's instructions (Promega, Madison, WI, USA). Briefly, the eye cups isolated from the anesthetized mice were fixed with 4% PFA overnight at 4 °C. The eye cups were digested with 20 μg/mL proteinase K for 10 min, and then incubated with the equilibration buffer for 10 min at room temperature. The samples were incubated with the rTdT incubation buffer for 60 min at 37 °C, and the reaction was terminated by using the SSC solution. The retinas were incubated with DAPI, and then mounted with the GCL being upturned. TUNEL signals were visualized with a confocal laser-scanning microscope through a 20 × objective (FluoView 1000, Olympus, Monolith, Tokyo, Japan). Serial deep scannings were done only in the GCL according to the DAPI staining. All TUNEL-positive signals that merged well with DAPI in each retina were counted.

### Flow cytometry assay

The retinas obtained from the anesthetized MacGreen mice were digested in the enzyme buffer containing 1 mg/mL collagenase D (#11088858001, Roche, Switzerland) and 30 U/mL DNase I (#18047019, Thermo Fisher, USA) in Hank’s balanced salt solution (HBSS) for 60 min at 37 °C, during which gentle shake was made every 15 min. Retinal cells were carefully dissociated using the fire-polished pipette and filtered through a 70 μm cell strainer (#352350, Corning, USA). After washing with the HBSS, cell suspension was centrifuged at 300 *g* for 5 min, and then the pellet was re-suspended in 100 µL stain buffer (#554657, BD Pharmingen, USA) containing Alexa Fluor 647-conjugated antibody for CD206 (#565250, BD Biosciences, USA), phycoerythrin-conjugated antibody for CD86 (#553692, BD Biosciences, USA), and incubated for 30 min on ice. Retinal microglia carrying GFP signal can be detected by fluorescence-activated cell sorting (FACS) (BD FACSCalibur, BD, USA) and analyzed by FlowJo V10.0 software (TreeStar, USA).

### RNA extraction and qPCR

Total RNA was extracted from each retina, primary cultured microglia and Müller cells using the AllPrep DNA/RNA/Protein Mini Kit Print (#80004, Qiagen, Hilden, Germany) or the TaKaRa MiniBEST Universal RNA Extraction Kit (#9767, Takara, Kusatsu, Japan), respectively. RNA was reverse transcribed using the PrimeScript™ RT reagent Kit with gDNA Eraser (#RR047A, Takara). mRNA was amplified using the TB Green^®^ Premix Ex Taq™ II (#RR820A, Takara, Japan). Forward and reverse primer sequences used in this study are listed in Additional file 1: Table S1. The thermal cycling conditions were 95 °C for 1 min, 40 cycles of 20 s at 95 °C, 30 s at 58 °C, and 40 s at 72 °C. β-actin was used as an internal control. Quantitative real-time polymerase chain reaction (qPCR) assays were performed on the QuantStudio 3 Real-Time PCR system (Thermo Fisher Scientific, Rockford, IL, USA), and the data were exported for analysis using 2^−ΔΔct^ calculation method.

### Enzyme-linked immunoabsorbent assay

Primary cultured microglia were treated with 100 μM BzATP for different times, and the culture mediums were collected, centrifuged, and stored at − 80 °C. The concentrations of TNF-α, IL-6, IL-4 and IL-10 were measured using the flowing enzyme-linked immunoabsorbent assay (ELISA) kits, respectively, according to the manufacturers’ instructions: anti-mouse-TNF-α ELISA kits (#MTA00B, R&D Systems, USA), anti-mouse-IL-6 ELISA kits (#M6000B, R&D Systems, USA), anti-mouse-IL-4 ELISA kits (#EM003, Genetimes Technology, China) and anti-mouse-IL-10 ELISA kits (#EM005, Genetimes Technology, Shanghai, China).

### Calcium imaging

Changes in intracellular calcium concentrations ([Ca^2+^]_i_) of microglia were assessed using the ratiometric dye Fura-2 AM, as described in detailed previously [16]. The retinas, obtained from the anesthetized MacGreen mice, were digested in 25 mL oxygenated HBSS containing 0.11 g papain (#2886967, Millipore, USA) and 0.025 g l-cysteine (#C7352, Sigma-Aldrich, USA) for 30 min at 34 °C. Retinal cells were mechanically dissociated with fire-polished Pasteur pipettes, and cell suspension was dropped onto the poly-d-lysine pre-coated coverslips in 24-well plates. Isolated retinal cells were cultured for 2–3 h under 37 °C/5% CO_2_, circumstance, and then incubated with 4 μg/mL Fura-2 (#F1221, Thermo Fisher, USA) in HBSS at 37 °C for 30 min. After washing twice in HBSS, the coverslips were transferred onto imaging system and continually perfused with Ringer’s solution containing (mM): NaCl 135, KCl 3, CaCl_2_ 2, MgCl_2_ 1, HEPES 10, glucose 11 and sucrose 10, pH adjusted to 7.4 with NaOH, 310 mOsm/L. Digital fluorescent images at both wavelengths (340 and 380 nm excitation) were captured every 5 s from the GFP-positive microglia with a Leica DMI3000 B microscope (Leica, Germany) equipped with a Cool SNAP HQ^2^ system (Photometrics, USA) at room temperature through a × 20/0.5 objective lens and an emission filter (510–550 nm). After stable recordings were reached, BzATP (300 μM) was applied to activate P2X7R for 350 s. The ratios of emitted fluorescence intensities at excitation wavelengths of 340 nm and 380 nm (F_340/F380_) were calculated and analyzed using the Metafluor software (Molecular Devices, USA).

### Statistical analysis

Statistical analysis was performed using GraphPad Prism (version 6.02, Graphpad Software Inc., La Jolla, CA, USA). Data were expressed as mean ± SEM. A one-way analysis of variance (ANOVA) with Dunnett’s multiple comparisons, Tukey–Kramer test (comparisons between two groups) and *t*-test (paired data) were appropriately used. *P* values of less than 0.05 were considered statistically significant in all tests.

## Results

### Dynamic changes of Müller cell and microglia activation in COH retinas

**Fig. 1:**
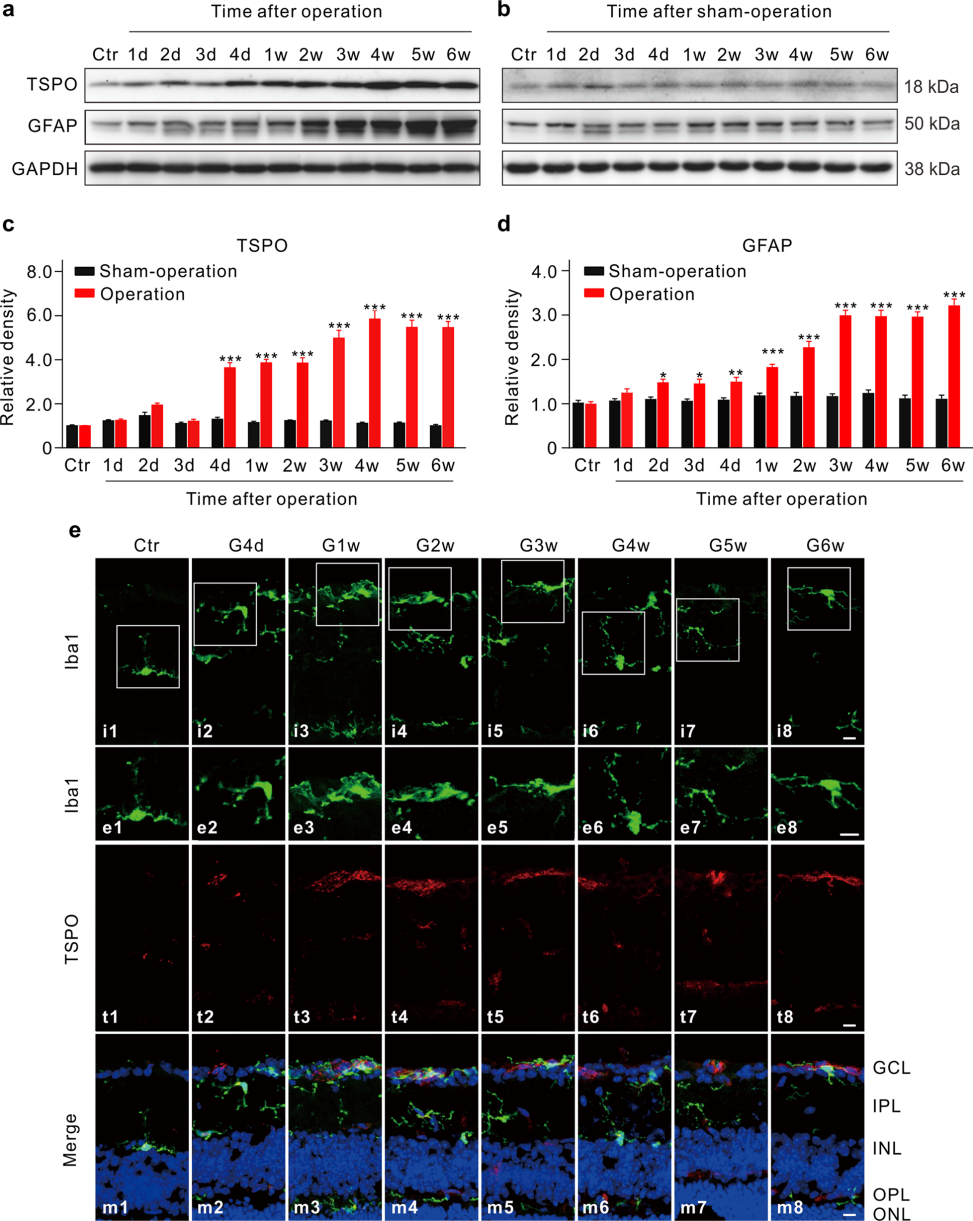
Dynamic changes in expression of GFAP and TSPO in mouse COH retinas. **a**, **b** Representative immunoblots obtained at different times after operation/sham-operation showing the changes of TSPO and GFAP expression in COH (**a**) and sham-operated (**b**) retinas. **c**, **d** Bar charts comparing the average relative densities of immunoreactive bands of TSPO (**c**) and GFAP (**d**) expression obtained at corresponding post-operational times in COH and sham-operated retinas. All the data are normalized to their corresponding GAPDH and then to the data before operation (control, Ctr). *n* = 6 for each group. *P < 0.05, **P < 0.01, and ***P < 0.001 vs. the data from the sham-operated group using paired two-tailed Student’s t test (**c**, **d**). **e** Photographs of immunofluorescence staining showing the morphological changes, migration of Iba1-labeled microglia (i1-8) and TSPO expression (t1-8) in retinal vertical slices taken from sham-operated mice (Ctr), and those obtained at different post-operational times (G4d, G1w, G2w, G3w, G4w, G5w, and G6w). e1-8 are enlarged images of the areas encircled by squares in i1-8, and m1-8 are merged images of i1-8 and t1-8. Scale bar: 10 μm for all images. *GCL* ganglion cell layer, *IPL* inner plexiform layer, *INL* inner nuclear layer, *OPL* outer plexiform layer, *ONL* outer nuclear layer

We first examined the dynamic changes of Müller cell and microglia activation in the mouse model of COH that was produced by injecting micro-magnetic beads into the anterior chamber [12, 13]. Changes of IOP in COH mice are shown in Additional file 1: Fig. S1. Figure 1a–d shows the changes in expression levels of GFAP and TSPO, markers for activated Müller cells and microglia, respectively, obtained at different times after the COH operation. A sharp and notable (3 ~ sixfold) increase in TSPO expression was observed at the fourth post-operational day (G4d) and the expression was kept at such higher levels through the sixth week (G6w) (Fig. 1a-c). The expression levels of GFAP were also increased with time, but exhibited a somewhat different course. That is, the expression level was mildly increased as early as at G2d and then reached a higher plateau at G3w in a progressive manner (Fig. 1a, b, d). The activation of microglia was accompanied by morphological changes. As shown in Fig. 1e (enlarged images) most clearly, the activated microglia displayed a ramified and amoeboid-like shape with enlarged somata, in contrast to branched microglia with smaller somata in normal retina. In addition, microglia were translocated from the inner/outer plexiform layer in control retinas to the GCL in COH retinas (Fig. 1e). Müller cells did not show detectable morphological changes in COH retinas (Additional file 1: Fig. S2).

### Activated Müller cells strengthen microglia activation through ATP/P2X7R pathway

**Fig. 2:**
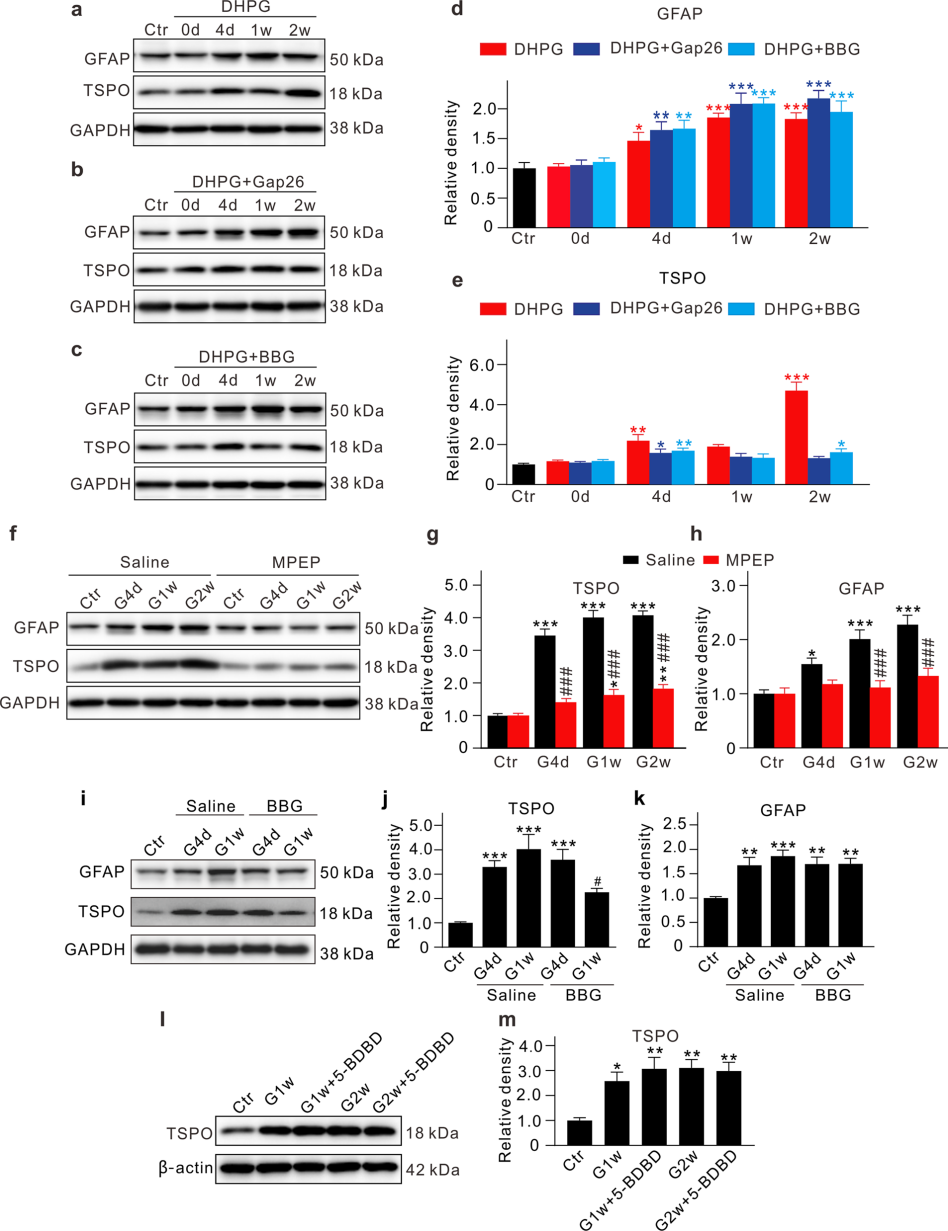
ATP released from activated Müller cells induces microglia activation through P2X7R. **a–c** Representative immunoblots showing the changes of GFAP and TSPO expression in retinas with intravitreal injections of DHPG (100 μM, 2 μL) (**a**), DHPG along with Gap26 (200 μM, 2 μL) (**b**), and DHPG along with BBG (10 μM, 2 μL) (**c**) at different times after the injections. **d**, **e** A comparison of average relative densitometric quantifications of the immunoreactive bands obtained following different kinds of injection is shown in the bar charts (**d** for GFAP; **e** for TSPO). **f** Representative immunoblots showing the changes of GFAP and TSPO expression at different post-operational times in normal and COH retinas with intravitreal injections of saline (2 μL)/20 μM MPEP (2 μL). Saline or MPEP injections were made two days before COH operation. **g**, **h** A comparison of average relative densitometric quantifications of the immunoreactive bands obtained following different kinds of injection is shown in the bar charts (**g** for TSPO; **h** for GFAP). **i** Representative immunoblots showing the changes in TSPO and GFAP protein expression in COH retinas with or without the intravitreal injection of BBG. 2 μL BBG (10 μM) or normal saline was intravitreally injected 2 day before the COH operation. **j**, **k** A comparison of average relative densitometric quantifications of the immunoreactive bands obtained following different kinds of injection is shown in the bar charts (**j** for TSPO; **k** for GFAP). **l**, **m** Western blot results showing the changes in TSPO protein expression in COH retinas with or without the intravitreal injection of 5-BDBD, a P2X4R antagonist. All the data are normalized to control. In all experiments, *n* = 6 for each group. *P < 0.05, **P < 0.01, and ***P < 0.001 vs. Ctr using ordinary one-way ANOVA with Dunnett’s multiple comparisons test (**d**, **e**, **g**, **h**, **j**, **k**, **m**); ^#^*P* < 0.05 and ^###^*P* < 0.001 vs. corresponding saline group at the same time point using paired two-tailed Student’s *t* test (**g**, **h**, **j**, **k**)

Since activated Müller cells release ATP mainly through Cx43 hemichannels [21–24], it raised a possibility that ATP released by activated Müller cells may induce microglia activation. To test this possibility, we first tried to understand what would happen to microglia by using intravitreal injections of DHPG, a group I metabotropic glutamate receptor (mGluR I) agonist, in normal mice, a procedure that has been shown to induce Müller cell activation through activating the mGluR5 subtype of mGluR I [14, 15]. Even though microglia also express mGluR I [25], it was unlikely that mGluR I was involved in microglia activation, because DHPG did not change the expression of TSPO in microglia in culture (Additional file 1: Fig. S3). In the DHPG-injected normal retinas, TSPO expression was progressively increased in the first week and then displayed a dramatic increase (Fig. 2a, e), while GFAP expression was slowly increased during the entire period (Fig. 2a, d). The time course of changes in TSPO expression induced by DHPG injection was slightly different from that of IOP elevation at the early stages. Co-injection of the Cx43 specific blocker Gap26 largely reduced the expression levels of TSPO in microglia obtained at different times after the co-injection (Fig. 2b, e). It is noteworthy that the TSPO expression level slightly increased with increasing time. Mouse retinal microglia are known to express two purinergic receptor subtypes, P2X7R and R2X4R, but only P2X7R was reported to be involved in RGC injury [26, 27]. To test whether P2X7R was involved in microglia activation, we examined effects of brilliant blue G (BBG), an antagonist of P2X7R, on TSPO expression in microglia in DHPG-injected retinas. As shown in Fig. 2c, e, co-injection of BBG along with DHPG largely reduced the TSPO expression levels obtained at different times.

To understand how Müller cell activation induced in COH retinas could influence microglia activation, TSPO expression levels in microglia were determined when Müller cell activation was suppressed by intravitreal pre-injection of MPEP, an antagonist of mGluR5. The results showed that TSPO expression levels became lower as GFAP expression levels were reduced (Fig. 2f–h). It should be noted that the expression level of TSPO was significantly increased as early as at G4d in COH retinas and then tended to level off, which is quite different from that observed in DHPG-injected normal retina. When MPEP was injected, the expression levels at G4d, G1w, and G2w were all largely decreased, but they were still higher than the levels before the injection. Moreover, pre-injection of BBG reduced the TSPO levels at G1w, but not the GFAP expression levels (Fig. 2i–k). In addition, intravitreal or intraperitoneal pre-injection of BBG also attenuated the morphological changes of microglia in COH retinas (Additional file 1: Fig. S4a, b). However, intravitreal pre-injection of 5-BDBD, a selective P2X4R antagonist, did not influence the expression of TSPO in COH retinas (Fig. 2l, m). All these results strongly suggest that ATP released due to Müller cell activation may activate microglia through P2X7R in COH mouse retinas.

**Fig. 3:**
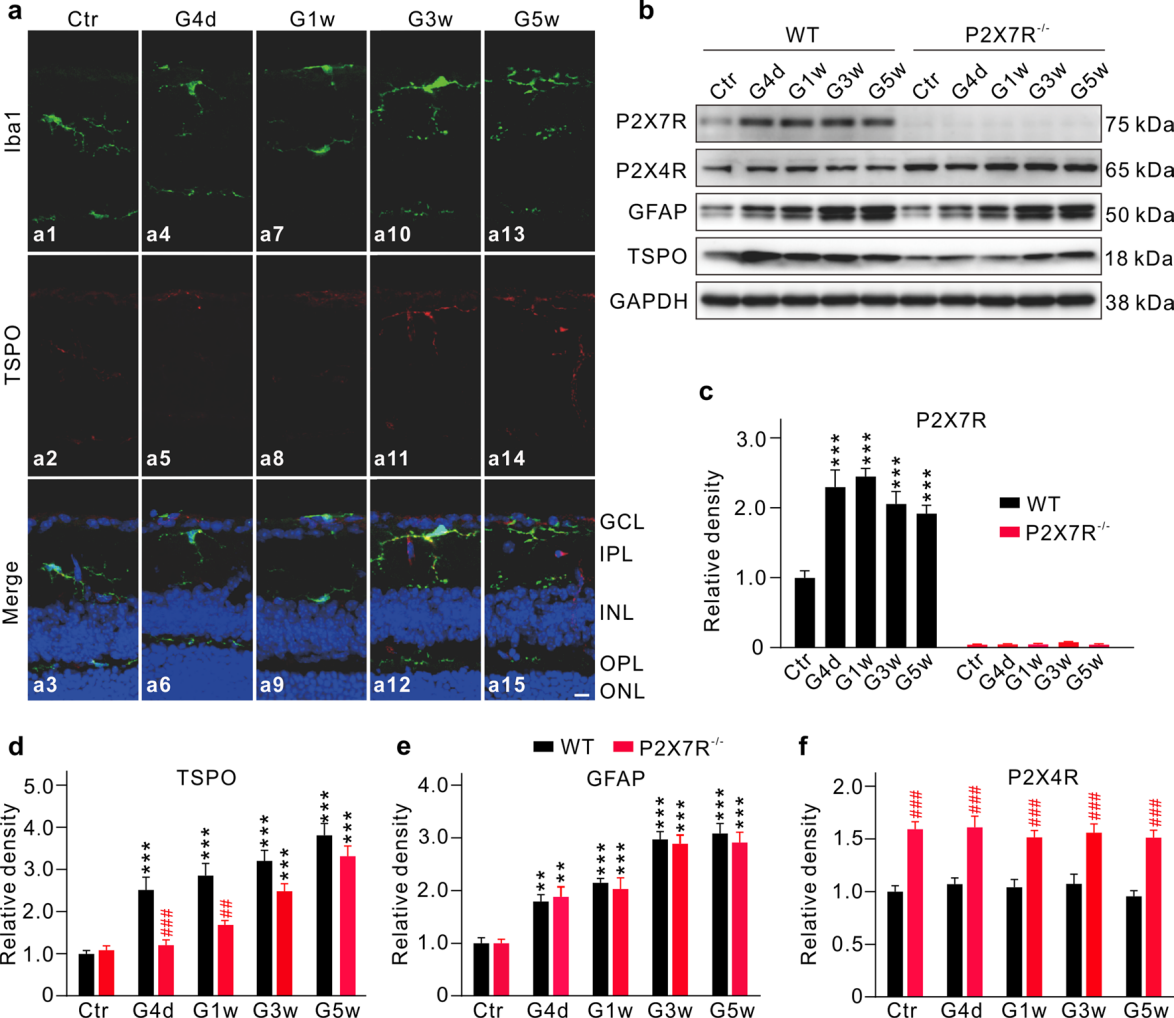
P2X7R-mediated changes of microglia in COH retinas. **a** Immunofluorescence staining showing changes in morphology and migration of the Iba1-labeled microglia, and in TSPO expression in retinal vertical slices taken from sham-operated *P2X7R*^−/−^ mouse (Control, Ctr), and *P2X7R*^−/−^ mice obtained at different post-operational times (G4d, G1w, G3w, and G5w). Scale bar: 10 μm for all images. **b** Representative western blotting results showing the changes in protein levels of P2X7R, P2X4R, TSPO, and GFAP in Ctr and COH retinas obtained from WT and *P2X7R*^−/−^ mice at different post-operational times. **c**–**f** Bar charts comparing the average relative densities of immunoreactive bands of P2X7R (**c**), TSPO (**d**), GFAP (**e**) and P2X4R (**f**) expression obtained at corresponding post-operational times in COH retinas of wild type and *P2X7R*^−/−^ mice. All the data are normalized to the corresponding controls. n = 6 for each group. *P < 0.05, **P < 0.01, and ***P < 0.001 vs. Ctr using ordinary one-way (**c**) and two-way (**d**, **e**) ANOVA with Dunnett’s multiple comparisons test; ^#^*P* < 0.05, ^##^*P* < 0.01, and ^###^*P* < 0.001 vs. corresponding WT mice at same time point using paired two-tailed Student’s *t* test (**d**, **f**). *GCL* ganglion cell layer, *IPL* inner plexiform layer, *INL* inner nuclear layer, *OPL* outer plexiform layer, *ONL* outer nuclear layer

We further tested changes in microglia morphology, TSPO expression, and the migration of microglia to the GCL induced by IOP elevation in P2X7R knockout (*P2X7R*^*−/−*^) mice immunohistochemically. Overall, these changes became less significant (Fig. 3a) as compared to those induced by IOP elevation in wild-type (WT) mice (Fig. 1e). Western blot analysis yielded similar results. GFAP levels at different times were not much changed from those determined in WT mice, which were increased with time (Fig. 3b, e). The changes in TSPO protein levels were different. Although the levels in COH *P2X7R*^*−/−*^ mice were remarkably lower than those of WT mice, they were steadily increased with time (Fig. 3b, d). As expected, the expression of P2X7R could be hardly detected in COH *P2X7R*^*−/−*^ mice (Fig. 3b, c). It should be noted, however, that the expression level of P2X4R was significantly enhanced (Fig. 3b, f).

**Fig. 4:**
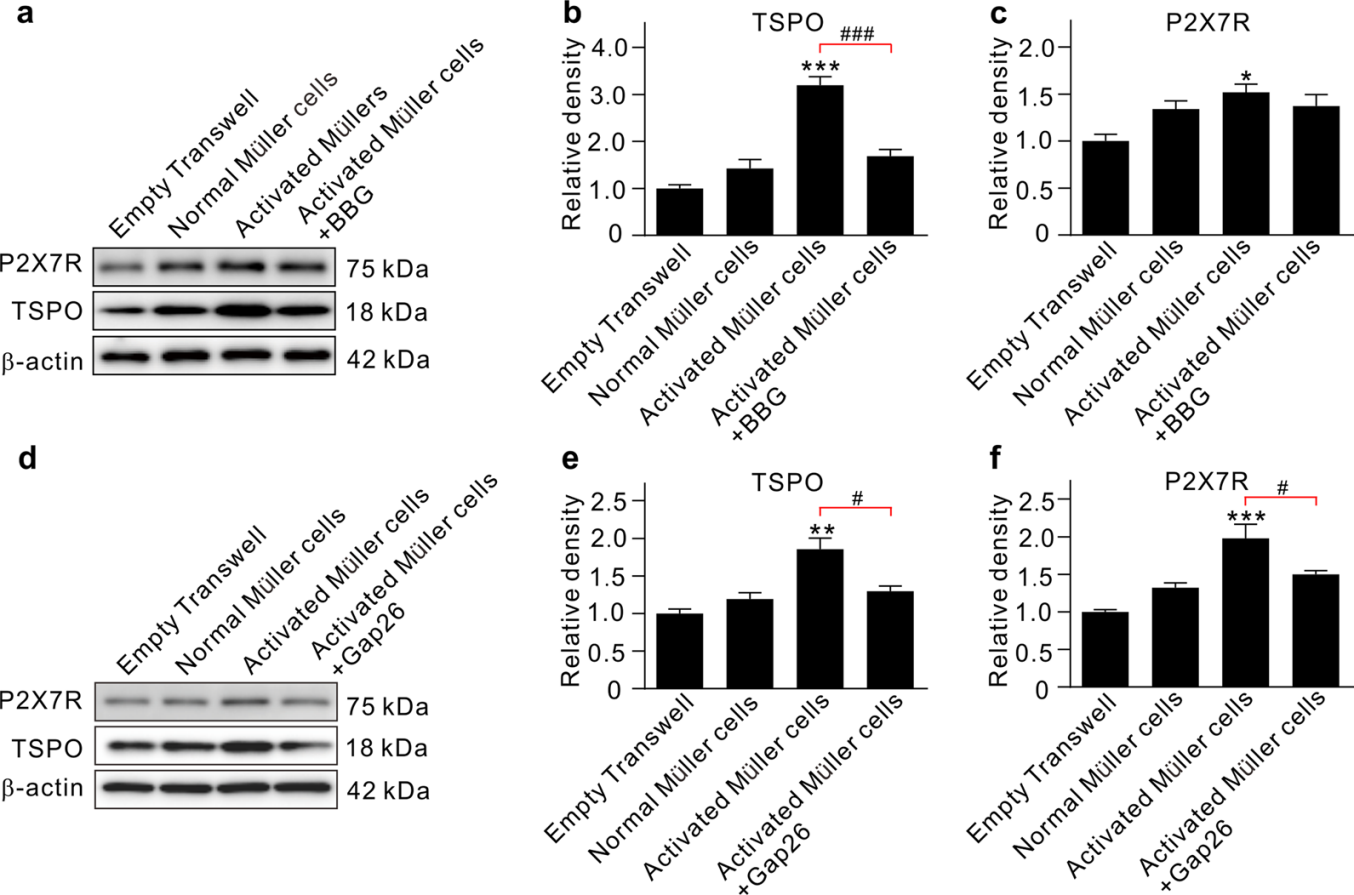
Activated Müller cells cause microglia activation via ATP/P2X7R pathway. **a** Representative immunoblots showing the changes in TSPO and P2X7R protein expression in cultured microglia which were co-cultured with normal or pre-activated Müller cells in the absence or presence of the P2X7R blocker BBG. Müller cells were pre-activated by incubating with DHPG for 72 h. BBG (10 μM) was added to culture medium of microglia 2 h before the activated Müller cells were added to the Transwell system. **b**, **c** A comparison of average relative densitometric quantifications of the immunoreactive bands obtained following different kinds of treatment is shown in the bar charts (**b** for TSPO; **c** for P2X7R). **d** Representative western blotting results showing the changes in protein levels of TSPO and P2X7R in cultured microglia which were co-cultured with normal or pre-activated Müller cells in the absence or presence of the Cx43 blocker Gap62. Gap26 (200 μM) was added to culture medium of Müller cells. **e**, **f** A comparison of average relative densitometric quantifications of the immunoreactive bands obtained following different kinds of treatment is shown in the bar charts (**e** for TSPO; **f** for P2X7R). All the data are normalized to empty transwell group. *P < 0.05, **P < 0.01, and ***P < 0.001 vs. empty transwell group using ordinary one-way ANOVA with Dunnett’s multiple comparisons test (**b**, **c**, **e**, **f**); ^#^*P* < 0.05 and ^###^*P* < 0.001 using unpaired two-tailed Student’s t test against the activated Müller cells group (**b**, **c**, **e**, **f**). All in vitro experiments: *n* = 3 biological replicates × 3 technical replicates

Transwell in vitro experiments using purified primary cultured Müller cells and microglia (Additional file 1: Fig. S5) were performed to further demonstrate the activated Müller cell-mediated microglia activation. When the Müller cells that were first activated by DHPG treatment were co-cultured with normal microglia for 72 h in the Transwell system, the expression levels of both TSPO and P2X7R in microglia were significantly increased. The enhanced expression levels were largely reduced by co-applying either BBG (Fig. 4a–c) or Gap26 (Fig. 4d–f).

### Interplay between Müller cells and microglia aggravates retinal inflammatory response

**Fig. 5:**
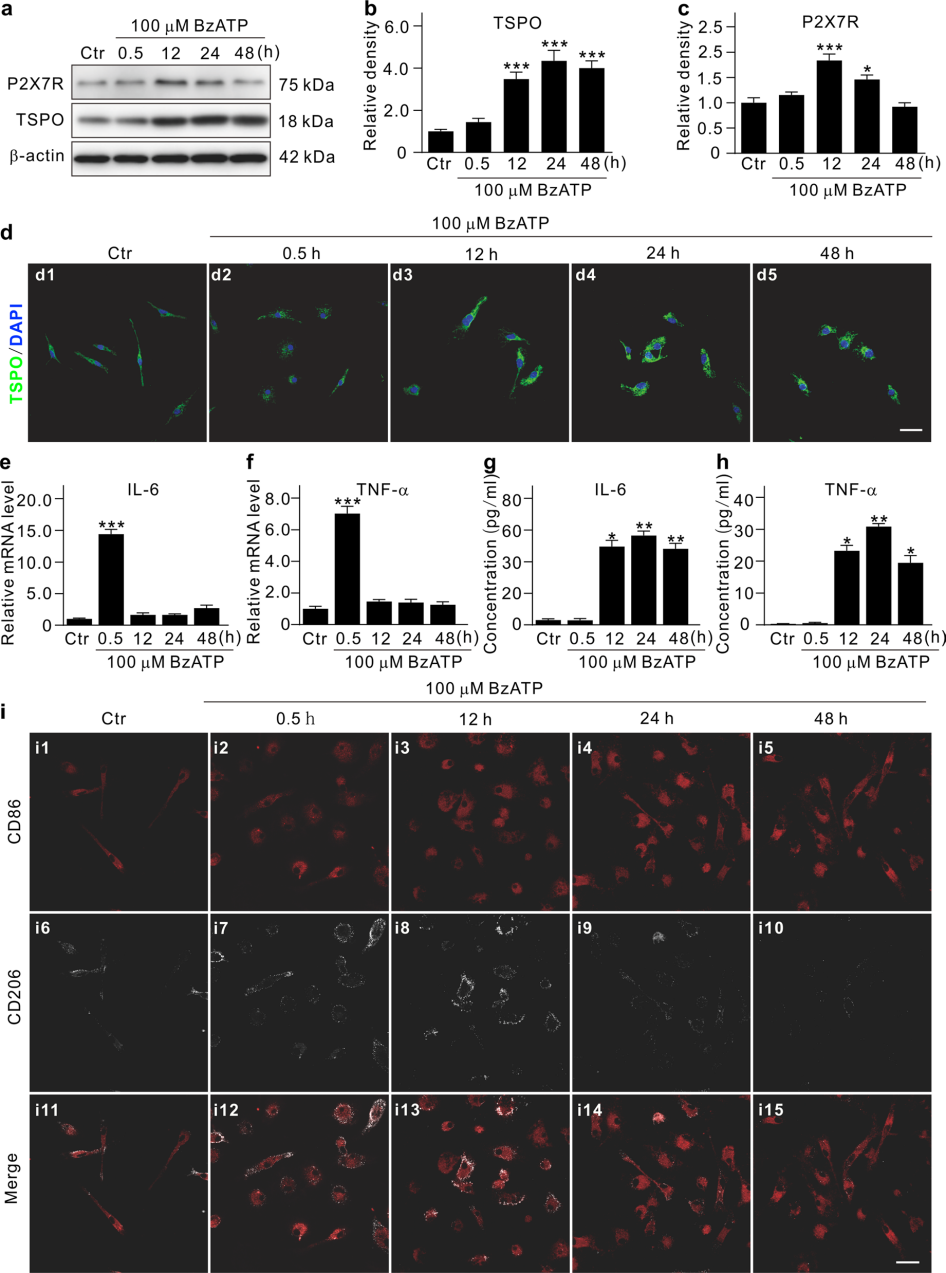
Pro-inflammatory factor release and dynamic changes of M1-like and M2-like genotypes of activated microglia. **a** Representative immunoblots showing the changes in TSPO and P2X7R protein expression in primary cultured microglia which were treated with 100 μM BzATP for different times. **b**, **c** A comparison of average relative densitometric quantifications of the immunoreactive bands obtained at different times of treatment is shown in the bar charts (**b** for TSPO; **c** for P2X7R). **d** Immunofluorescent labeling showing the changes in morphology and TSPO expression in primary cultured microglia after the cells were treated with 100 μM BzATP for different periods of time. Scale bar: 20 μm for all images. **e, f** Cumulative data summarizing the changes in mRNA levels of IL-6 (**e**) and TNF-α (**f**) in cultured microglia extracts obtained in Ctr and those with BzATP treatment for different periods of time. **g, h** Bar chart showing the average extracellular IL-6 (**g**) and TNF-α (**h**) concentrations in cultured microglia in Ctr and groups of BzATP treatment for different periods of time. **i** Double immunofluorescent labeling showing the dynamic changes in expression of CD86 and CD206 in primary cultured microglia after the cells were treated with 100 μM BzATP for different periods of time. Scale bar: 20 μm for all images. *P < 0.05, **P < 0.01, and ***P < 0.001 vs. Ctr using ordinary one-way ANOVA with Dunnett’s multiple comparisons test (**b**, **c**, **e**–**h**). All in vitro experiments: *n* = 3 biological replicates × 3 technical replicates

Possible functional significance of activated microglia was examined. BzATP, a specific P2X7R agonist, was used to activate primary purified cultured microglia for different times. Western blot analysis showed that the TSPO protein levels were increased with time following BzATP application, reaching a plateau in 12 h (Fig. 5a, b). BzATP application also induced morphological changes of microglia (Fig. 5d, Additional file 1: Fig. S6a). In addition, the P2X7R protein levels in microglia were also increased during the initial stage (< 12 h) of BzATP treatment, but then decreased to the control level in a progressive manner (Fig. 5a, c). Microglia are commonly categorized into two opposite phenotypes: M1-like and M2-like. Activation of these two phenotypes may generate pro-inflammatory (for M1-like) or anti-inflammatory (for M2-like) effects, respectively [28–30], through the production of pro-inflammatory factors, such as IL-6, TNF-α, and inducible nitric oxide synthase (iNOS), and the secretion of anti-inflammatory factors, such as IL-4 and IL-10, respectively. Using qPCR assay, it was found that the mRNA levels of IL-6, TNF-α, and iNOS were largely increased immediately at the early stage of BzATP application (in 0.5 h) (Fig. 5e, f; Additional file 1: Fig. S6b), and both levels almost returned to the control levels at the later stages (in 12, 24, and 48 h). In contrast, the concentrations of IL-6 and TNF-α proteins in the culture medium of microglia, as assayed by ELISA, did not change at the early stages of BzATP application and started to increase in 12 h and were thereafter maintained at such higher levels (Fig. 5g, h). Although the mRNA levels of IL-4 and IL-10 were also increased (Additional file 1: Fig. S6c-d), the protein concentrations were undetectable by using the ELISA kits. Immunocytochemical experiments demonstrated that the number of CD86^+^ M1-like microglia was gradually increased during the entire period of BzATP treatment, whereas the number of CD206^+^ M2-like microglia was increased only at the early stages of BzATP treatment (0.5–12 h) (Fig. 5i).

**Fig. 6:**
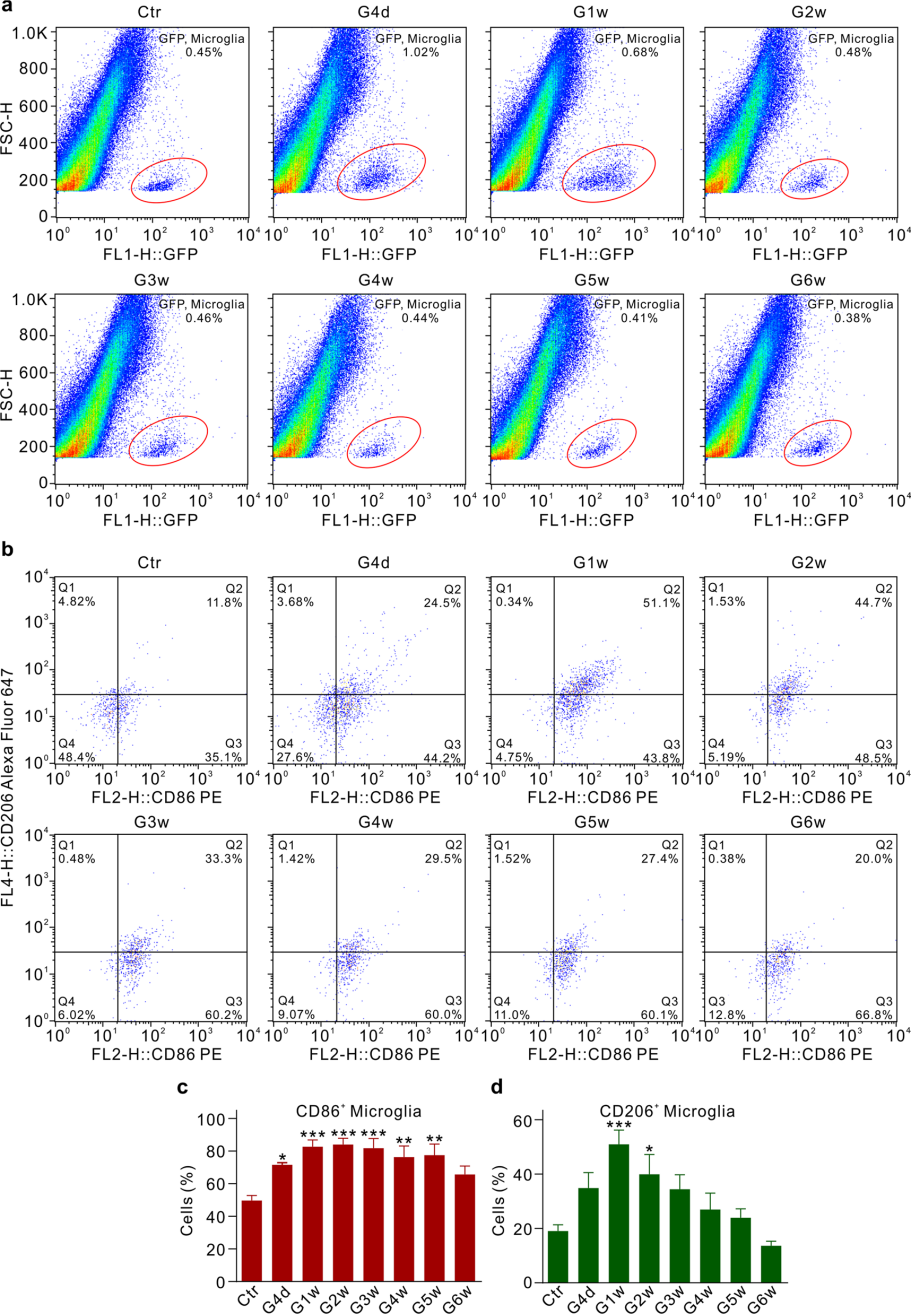
Dynamic changes of M1-like and M2-like phenotype microglia in COH retinas of the MacGreen transgenic mice. **a** Representative fluorescence activating cell sorter (FACS) plots of retinal cells obtained from sham-operated (control, Ctr) and COH MacGreen mice at different post-operational times, assayed by flow cytometry. Microglia in the MacGreen mice express the enhanced green fluorescent proteins. **b** Representative gating strategy for identifying CD206^+^ and CD86^+^ microglia in retinal cells obtained from Ctr and COH MacGreen mice at different post-operational times. **c**, **d** Bar charts showing the changes in the percentage of CD86^+^ M1-like (**d**) and CD206^+^ M2-like (**e**) microglia in whole retinal cells obtained from Ctr and those of COH retinas of the MacGreen mice different post-operational times. *n* = 6 for each group. *P < 0.05, **P < 0.01, and ***P < 0.001 vs. Ctr using ordinary one-way ANOVA with Dunnett’s multiple comparisons test (**c**, **d**)

Dynamic changes of M1-like and M2-like microglia were further analyzed in COH retinas of MacGreen transgenic mice, in which the green fluorescent protein (GFP) was highly expressed in retinal microglia, using flow cytometry techniques. Figure 6a, b shows representative fluorescence activating cell sorter (FACS) plots and gating strategy for identifying CD206^+^ and CD86^+^ microglia in retinal cells obtained from control and COH mice (from G4d through G6w). The percentage of CD86^+^ M1-like microglia was obviously increased as early as at G4d and then kept at higher levels through G6w (Fig. 6c), while the percentage of CD206^+^ M2-like microglia was transiently increased (between G1w and G2w) (Fig. 6d).

**Fig. 7:**
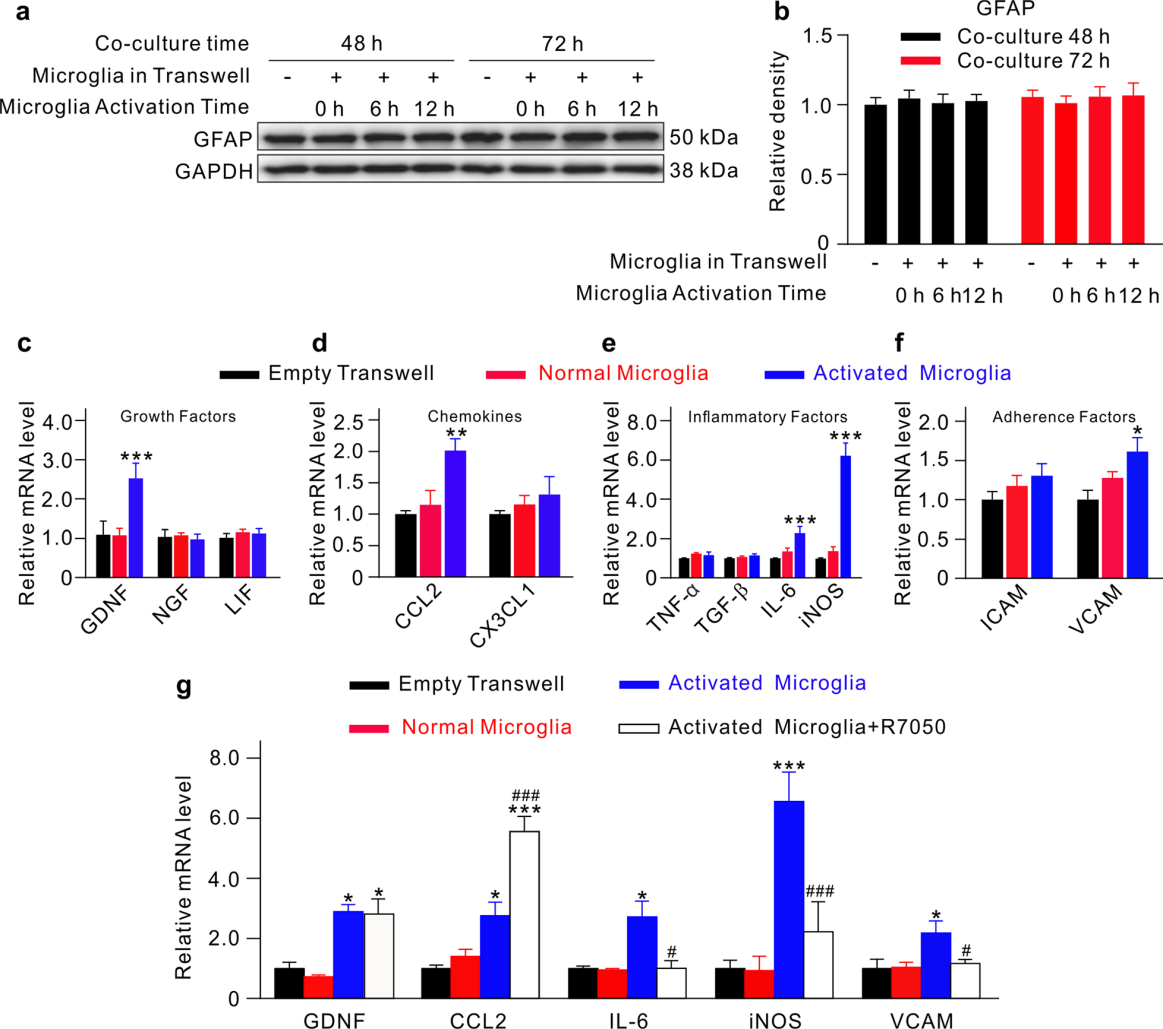
Activated microglia-induced functional changes in cultured Müller cells. **a** Representative Western blotting results showing the changes in GFAP protein expression in Müller cells which were co-cultured with pre-activated microglia by BzATP treatment for different times (0, 6, and 12 h). **b** A comparison of average relative densitometric quantifications of the immunoreactive bands of GFAP obtained following different kinds of treatment is shown in the bar charts. **c**–**f** Bar charts summarizing the changes in mRNA levels of growth factors (**c**), chemokines (**d**), inflammatory factors (**e**), and adherence factors (**f**) in Müller cells which were co-cultured in the Transwell system with normal or pre-activated microglia. **g** Bar charts summarizing the changes in mRNA levels of GDNF, CCL2, IL-6, iNOS, and VACM in Müller cells without or with the TNFR1 antagonist R7050. Müller cells were co-cultured in the Transwell system with normal, pre-activated microglia, and activated microglia + R7050. All the data are normalized to corresponding empty Transwell groups. *P < 0.05, **P < 0.01, and ***P < 0.001 vs. empty Transwell group using ordinary one-way ANOVA with Dunnett’s multiple comparisons test (**c**–**f**). ^#^*P* < 0.05, ^###^*P* < 0.001 vs. activated microglia group using unpaired two-tailed Student’s *t* test (**g**). All in vitro experiments above: *n* = 3 biological replicates × 3 technical replicates. *GDNF* glial cell line-derived neurotrophic factor, *NGF* nerve growth factor, *LIF* leukemia inhibitory factor, *CCL2* chemokine C–C-motif ligand 2, *CX3CL1* chemokine C-X3-C-motif Ligand 1, *TNF-α* tumor necrosis factor-α, *TGF-β* transforming growth factor-β, *IL-6* interleukin-6, *iNOS* inducible nitric oxide synthase, *ICAM* intercellular cell adhesion molecule, *VCAM* vascular cell adhesion molecule

How activated microglia affect Müller cells were further explored by Transwell in vitro experiments, which revealed the unchanged GFAP expression in Müller cells co-cultured with BzATP-activated microglia (Fig. 7a, b). However, the mRNA levels of glial cell line-derived neurotrophic factor (GDNF), chemokine C–C-motif ligand 2 (CCL2), IL-6, iNOS and vascular cell adhesion molecule (VCAM) in Müller cells were all significantly increased when co-cultured with activated microglia (Fig. 7c–f). The increases in mRNA levels of IL-6, iNOS, and VCAM were abolished by R7050, a specific TNFR1 antagonist (Fig. 7g).

### P2X7R/Ca^2+^/NFAT/NF-kB pathway mediates microglia inflammatory response

**Fig. 8:**
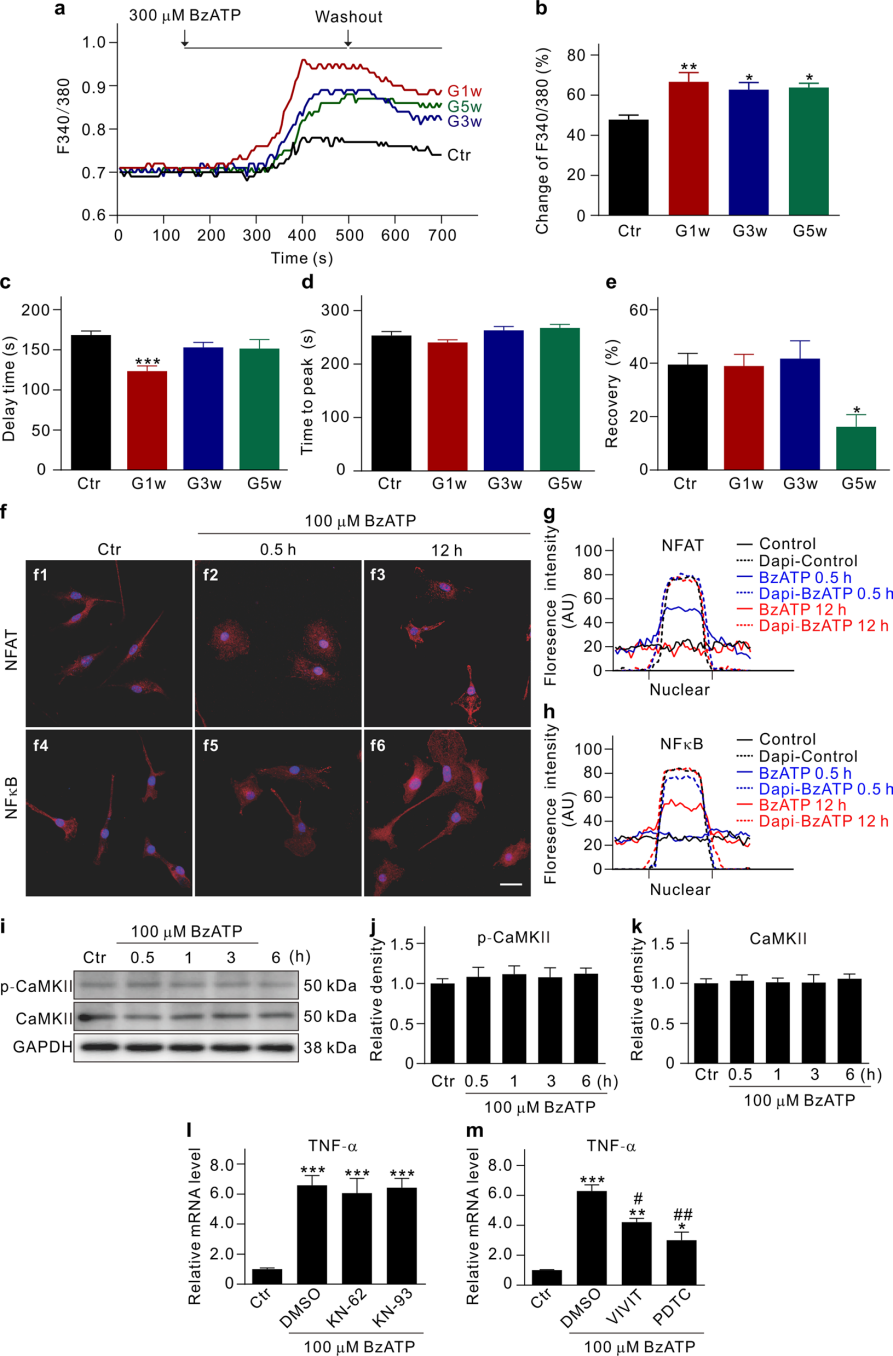
P2X7R/Ca^2+^/NFAT/NF-kB pathway mediates microglial inflammatory response. **a** BzATP-induced changes in intracellular Ca^2+^ concentrations ([Ca^2+^]_i_), indicated by F_340/380_, in microglial cells acutely isolated from control (Ctr) and COH retinas of the MacGreen mice at different post-operational times. **b**–**e** Bar charts summarizing the changes of peak value of F_340/380_ (**b**), delay time (**c**), time to peak (**d**), and recovery (**e**) in microglial cells of Ctr and cells of COH retinas at different post-operational times. *n* = 10 for each group. *P < 0.05, **P < 0.01, and ***P < 0.001 vs. Ctr using ordinary one-way ANOVA with Dunnett’s multiple comparisons test (**b**–**e**). **f** Immunofluorescent images showing the BzATP treatment-induced translocation of NFAT and NFkB from cytoplasm to nuclei. Scale bar: 20 μm for all images. **g**, **h** Quantitative analyses of the immunofluorescent densities of NFAT (**g**) and NF-kB (**h**) in microglial cells under different conditions. The lines show the average values of 10 cells in each group. Nucleus were stained with DAPI. **i** Representative immunoblots showing the expression of phosphorylated CaMKII (p-CaMKII) and total CaMKII in retinal primary cultured microglia with or without 100 μM BzATP treatment for different times. **j**, **k** A comparison of average relative densitometric quantifications of the immunoreactive bands obtained following different treatment times is shown in bar charts (**j** for p-CaMKII; **k** for total CaMKII). **l** Bar chart showing the changes in TNF-α mRNA levels induced by BzATP (100 μM) treatment for 30 min in primary cultured microglia with or without the presence of the CaMKII inhibitors KN62 and KN93. **m** Bar chart showing the changes in TNF-α mRNA levels induced by BzATP (100 μM) treatment for 30 min in primary cultured microglia with or without the presence of either the NFAT inhibitor VIVIT or the NF-kB inhibitor PDTC. *P < 0.05, **P < 0.01, and ***P < 0.001 vs. Ctr using ordinary one-way ANOVA with Dunnett’s multiple comparisons test (**l**, **m**); ^#^*P* < 0.05, and ^##^*P* < 0.01 vs. DMSO group using unpaired two-tailed Student’s *t* test (**m**). All in vitro experiments: *n* = 3 biological replicates × 3 technical replicates

P2X7R, a non-selective cation channel, is permeable to Ca^2+^, Na^+^, and K^+^ [31, 32]. To understand whether and how intracellular Ca^2+^ could mediate the release of the inflammatory factor TNF-α, transient intracellular Ca^2+^ concentrations ([Ca^2+^]_i_) induced by BzATP were measured in microglia acutely isolated from COH retinas of MacGreen mice. The peak values of fluorescence 340/380 (F_340/380_) were significantly increased in the cells of COH retinas from G1w to G5w, compared to the controls (Fig. 8a, b). In addition, although the time to peak of [Ca^2+^]_i_ after BzATP application was not much different from the control, the delay time in microglia at G1w and the recovery of [Ca^2+^]_i_ in microglia at G5w were decreased (Fig. 8c–e), suggestive of increased [Ca^2+^]_i_ in the cells of COH retinas. Since Ca^2+^-dependent TNF-α release could be mediated by calcineurin/NFAT and NF-kB pathways [19, 33], we investigated translocation of NFAT and NF-kB in cultured microglia and found that these factors were translocated from cytoplasm into nuclei at 30 min and 12 h, respectively, following the BzATP treatment (Fig. 8f–h). However, BzATP treatment did not affect calcium/calmodulin-dependent protein kinase II (CaMKII) and p-CaMKII protein levels (Fig. 8i–k). Inhibition of CaMKII activity had no effects on BzATP-induced increase in TNF-α mRNA levels (Fig. 8l), whereas inhibition of either NFAT or NF-kB by VIVT or PDTC blocked the BzATP-induced increase in TNF-α mRNA levels (Fig. 8m). These results indicate that the P2X7R/Ca^2+^/NFAT/NF-kB signaling pathway mediates microglia inflammatory response in COH retinas.

Finally, we explored effects of intravitreal injections of either MPEP or BBG, which inhibited Müller cell activation or P2X7R, on RGC apoptosis in COH retinas. The results showed that both MPEP and BBG significantly reduced the number of TUNEL-positive RGCs (Additional file 1: Fig. S7A, B), indicating that Müller cell activation and ATP/P2X7R-mediated microglia activation contribute to RGC apoptotic death in glaucoma.

## Discussion

Retinal microglia are activated by a variety of injurious signals, which is likely mediated by Toll-like receptors (TLRs) [5, 34]. Specifically, in human glaucomatous samples the expression of TLRs is upregulated in microglia, thus causing chronic microglia activation [34]. Since microglia make specific and direct contacts with normal neuronal synapses, these cells may execute either pro- or anti-inflammatory actions in a direct manner [27, 30, 35–37]. Moreover, it is known that microglia and Müller cells interact to play homeostatic roles in maintaining the milieu of the retina and such an interaction is involved in shaping retinal overall response to injury [4–6, 10, 11]. It has been generally thought that microglia react rapidly (within minutes) to an injurious signal [38]. However, our results provide evidence that activation of Müller cells may precede the activation of microglia, at least in COH retina. Since the TSPO expression in microglia was largely reduced when Müller cell activation was inhibited by MPEP or the ATP release was blocked by Gap26, or P2X7R was suppressed by BBG, it seems appropriate to conclude that the activation of microglia in COH retina was mostly, but not exclusively, mediated by Müller cell activation and the remaining part of the TSPO expression is speculated to be contributed by microglia activation directly induced by COH. This speculation was supported by the results reported in this work. As shown in Fig. 3, the expression levels of TSPO at G1w were largely reduced in COH retinas of *P2X7R*^*−/−*^ mice, as compared to the data obtained in wild-type mice. It is noteworthy that the TSPO levels tended to become higher with time thereafter so that the levels obtained at G5w were comparable to the values obtained in wild-type mice, which could be due to the compensatory effect mediated by increased expression of P2X4R in the knockout mice. Moreover, increased mRNA levels (TNF-α, iNOS, and IL-6) were seen when microglia were activated [27, 39, 40]. It seems likely that microglia activation in COH retinas may directly affect the functional states of retinal neurons by increasing the production of pro-inflammatory factors. In addition, Müller cell gliosis induced in COH retina could further activate microglia to aggravate inflammatory response. In laser-induced ocular hypertension (OHT) mice, major histocompatibility complex class II molecule (MHC-II) expression was upregulated both in activated Müller cells and microglia, which may involve in glaucoma pathophysiology [41–43]. While functional implication of direct and indirect actions of microglia activation remains to be further explored in the future, these results suggest a plentiful possibility that to suppress the interplay between Müller cells and microglia may ameliorate injury of RGCs under COH.

Another significant finding in the present work is that the signaling pathway mediating the interplay between Müller cells and microglia was revealed. Müller cells are a prominent source of extracellular ATP [21–24]. We still have no idea about the factors that are involved in inducing the release of ATP from Müller cells under COH. A possibility is that increased extracellular glutamate in COH retina induces ATP release from Müller cells by activating mGluR I since the mGluR I agonist DHPG treatment of primary cultured Müller cells resulted in increased levels of ATP in cultured medium through mGluR I/Gq/PI-PLC/PKC signaling pathway [23]. It should be noted that this signaling pathway is also involved in Müller cell activation in COH retina [14, 15], suggesting that ATP may be released from Müller cells at the activated state. Furthermore, both in vitro and in vivo experiments showed that Gap26, but not BBG, completely blocked the increased expression of TSPO in microglia, indicating that ATP released from Müller cells was mainly mediated by Cx43 in COH retina. ATP, in turn, induced microglia activation and promoted pro-inflammatory factor release through the ATP/P2X7R/Ca^2+^/NFAT/NF-kB pathway. On the other hand, ATP also induced a transient increase in mRNA levels of anti-inflammatory factors (IL-4 and IL-10) in microglia. However, the protein concentrations of anti-inflammatory factors were rather low, suggesting that neuroprotective effect of activated microglia could be ignored. In addition, activated microglia affect the functional conditions of Müller cells in a non-gliosis manner. The mRNA levels of GDNF and pro-inflammatory factors in Müller cells were significantly increased when these cells were co-cultured with the activated microglia. Increased expression of GDNF mRNA induced by activated microglia indicates that neuroprotective effect may be a consequence of the secondary response of Müller cells in glaucoma. Upregulation of pro-inflammatory factor mRNA expression suggests that the interaction of retinal glial cells may promote retinal inflammatory response in a positive feedback manner, which seemed likely to be mediated by inflammatory factors released from activated microglia [44–47]. Indeed, we observed that the TNFR1 inhibitor R7050 blocked the increase in mRNA levels of IL-6 and iNOS. It is noted that the mRNA levels of chemokine CCL2 and adhesion molecule VCAM in Müller cells were increased in response to activated microglia, which may increase protein expression on the surface of the cells. These molecules may involve in microglial translocation in glaucoma [10]. The detailed mechanisms remain to be explored.

In the CNS, activated microglia display M1-like and M2-like phenotypes, which produce cytotoxic and neuroprotective effect, respectively [28–30]. In this study, we found that retinal microglia underwent dynamic changes of M1-like and M2-like phenotypes in experimental glaucoma. Like seen in neurodegenerative diseases in the CNS [30], microglia in glaucomatous retinas showed mixed activation phenotypes. That is, M2-like microglia were activated only at the early stages of IOP elevation, while M1-like type microglia were sustainably activated during the whole period of IOP elevation (Figs. 5, 6), similar to the reports in mouse COH model, rd1 mouse model of retinal degeneration, and oxygen-induced retinopathy model [37, 48, 49]. These results suggest that activated microglia may provide a certain neuroprotective effect at the early stage of glaucoma, whereas the predominant effect of activated microglia is to induce retinal neuronal injury. Additionally, our results showed that a single microglia may express both CD206 and CD86, which suggests that individual microglia may undergo a switch from one phenotype to another during the process of glaucoma. The mechanisms underlying the phenotypic transformation of microglia remain to be explored in the future study.

Retinal glial activation-mediated inflammatory factor release contributes to RGC apoptosis in glaucoma [2–8, 50]. Activated retinal glial cells may be involved in RGC apoptosis at least through two pathways. The first pathway is that inflammatory factors released from either activated Müller cells or activated microglia induce RGC apoptosis directly. The second pathway is that ATP released from activated Müller cells induces microglia activation through P2X7R, thus promoting inflammatory factor release and RGC injury [27, 37, 51]. Interaction of activated Müller cells and microglia may further deteriorate retinal inflammatory response, and consequently aggravate RGC death.

## Conclusions

We provide robust evidence demonstrating that macroglia activation initiates the activation of retinal microglia in experimental glaucoma. Interaction of activated Müller cells and microglia aggravates retinal inflammatory response. Appropriate reduction of such an interaction may be a potential treatment strategy for preventing the loss of RGCs in glaucoma [27, 37, 51].

## Supplementary Information

**Additional file 1.**

## Data Availability

The data supporting the findings of this study are available within this article and its additional information files. All other relevant data are available from the corresponding authors upon reasonable request. Source data are provided with this paper.

## Abbreviations

BBG: Brilliant blue G
CaMKII: Calcium/calmodulin-dependent protein kinase II
CCL2: Chemokine C–C-motif ligand 2
COH: Chronic ocular hypertension
GDNF: Glial cell line-derived neurotrophic factor
GCL: Ganglion cell layer
GFAP: Glial fibrillary acidic protein
ILs: Interleukins
iNOS: Inducible nitric oxide synthase
IOP: Intraocular pressure
mGluR I: Group I metabotropic glutamate receptor
NO: Nitric oxide
P2X7R: P2X7 receptor
RGCs: Retinal ganglion cells
TNF-α: Tumor necrosis factor-α
TSPO: Translocator protein
TNFR1: TNF-α receptor 1
TUNEL: Terminal dUTP nick end labeling
VCAM: Vascular cell adhesion molecule
