# Supplementary material for: Interplay between Müller cells and microglia aggravates retinal inflammatory response in experimental glaucoma
Source: J Neuroinflammation. 2021 Dec 24;18:303. doi: 10.1186/s12974-021-02366-x (PMC8705189; doi:10.1186/s12974-021-02366-x)
Supplement: Supplementary file 1 — Additional file 1. [file 12974_2021_2366_MOESM1_ESM.pdf]

Supplementary Information for

Interplay between Müller cells and microglia aggravates retinal inflammatory response in experimental glaucoma

Xin Hu, Guo-Li Zhao, Meng-Xi Xu, Han Zhou, Fang Li, Yanying Miao, Bo Lei, Xiong-Li Yang*, Zhongfeng Wang*

*Corresponding authors. E-mail: zfwang@fudan.edu.cn (Z. WANG); xlyang@fudan.edu.cn (X.-L. YANG).

This PDF file includes:

Figures S1 to S7

Tables S1

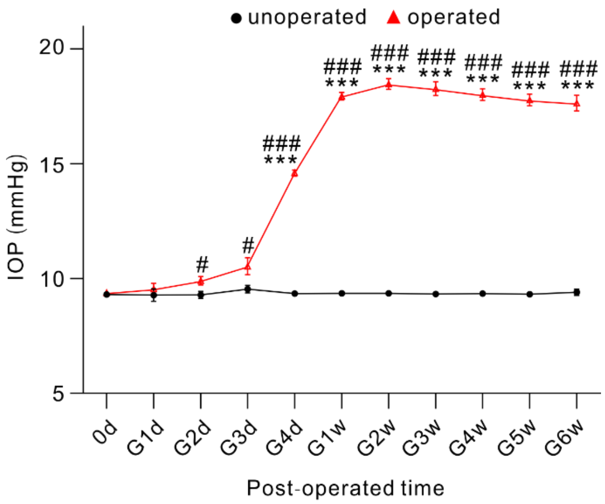

Fig. S1. Changes of IOP of both eyes in COH mice. Time course of IOP values of both eyes. The right eyes (operated) were injected the micro-magnetic beads. n = 11~392, $^{***}P < 0.001$ *vs*. 0d using ordinary one-way ANOVA with Dunnett's multiple comparisons test; $^{\#}P < 0.05$, and $^{\#\#\#}P < 0.001$ vs. unoperated eyes (left eyes) at the same time point using unpaired two-tailed Student's t test.

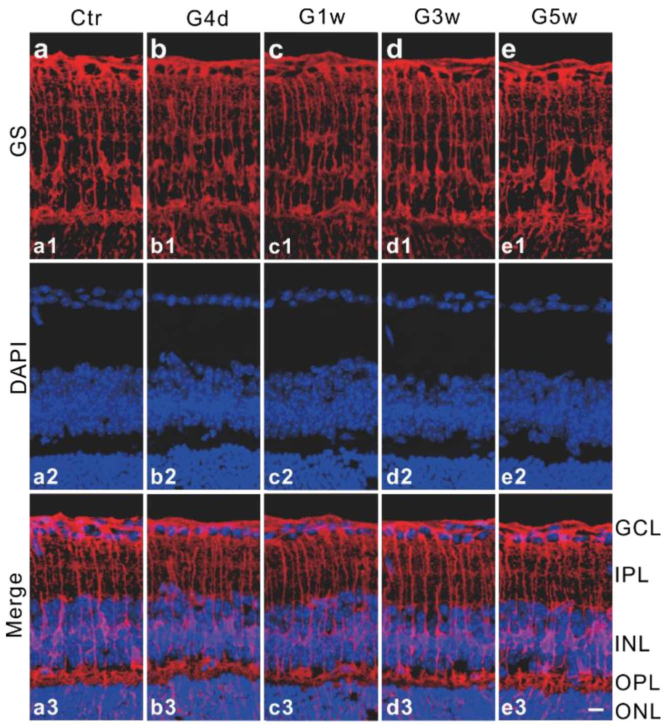

Fig. S2. No change of glutamine synthase expression in Müller cells of COH retinas. **(a-e)** Representative immunofluorescent labeling showing the expression of glutamine synthase (GS) in retinal vertical slices taken from sham-operated retina (Ctr) and COH retinas at different post-operational times (G4d, G1w, G3w, and G6w). n = 3 for each group. Nucleus were stained with DAPI. Scale bar: 10 μm for all images. GCL: ganglion cell layer; IPL: inner plexiform layer; INL: inner nuclear layer; OPL: outer plexiform layer; ONL: outer nuclear layer.

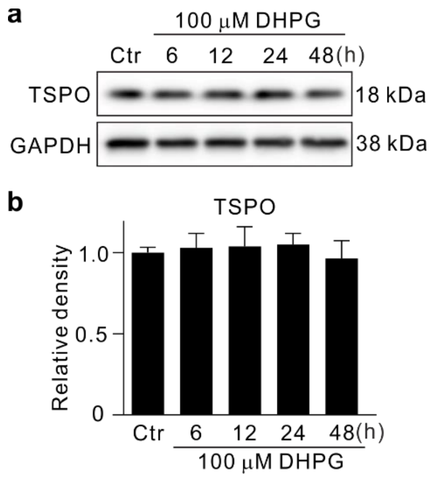

Fig. S3. Activation of mGluR I by DHPG does not induce microglia activation. (a) Representative immunoblots showing the changes of TSPO expression in retinal primary cultured microglia with 100 μM DHPG treatment for different times. **(b)** A comparison of average relative densitometric quantifications of the immunoreactive bands of TSPO expression obtained following different treatment times is shown bar charts. All the data are normalized to Ctr. No significant difference is found using ordinary one-way ANOVA with Dunnett's multiple comparisons test against Ctr. All *in vitro* experiments: n = 3 biological replicates × 3 technical replicates.

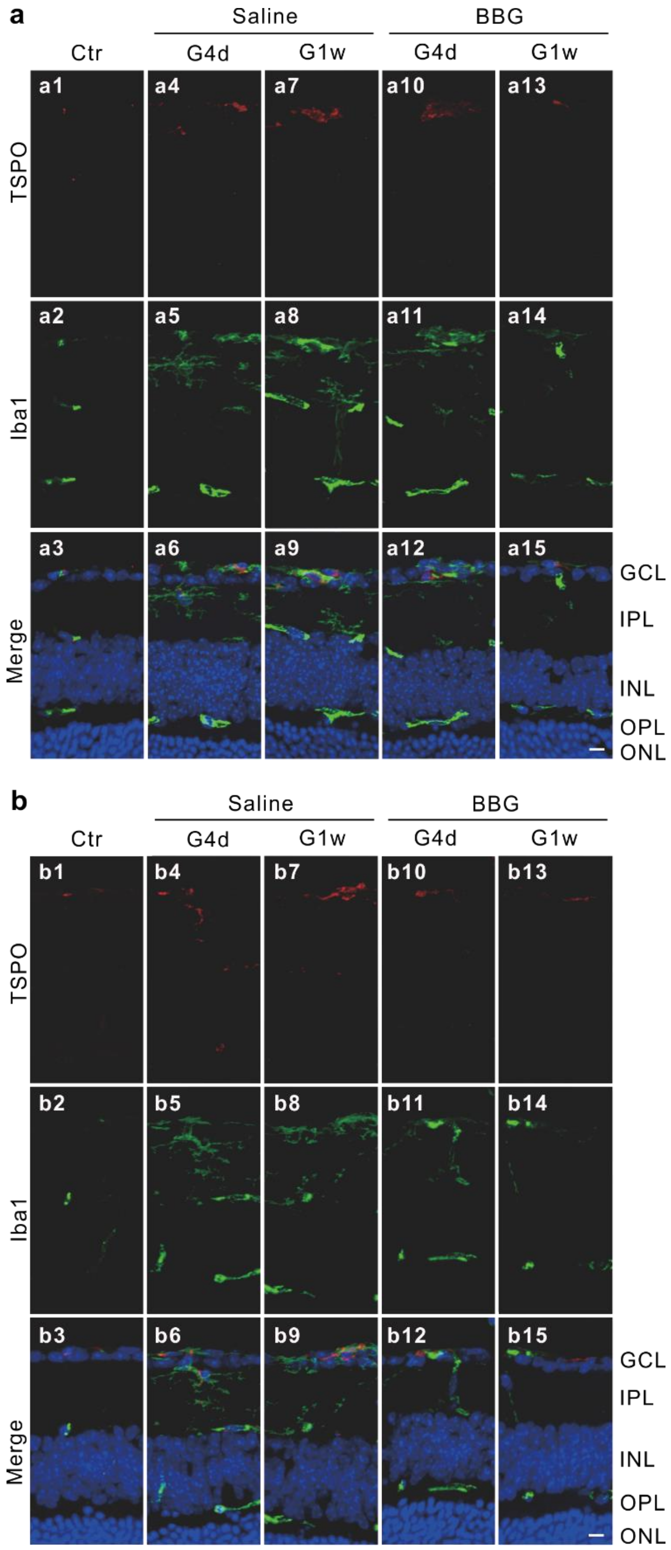

Fig. S4. Inhibition of P2X7R attenuates microglia activation in COH retinas. (a, b) Representative immunofluorescent labeling showing the changes in TSPO expression in retinal vertical slices taken from sham-operated retina (Ctr) and COH retinas at G4d and

G1w with or without injection of BBG. BBG was intravitreally injected two days before COH operation (a) or intraperitoneally injected every two days starting from the day before COH operation (b). Nucleus were stained with DAPI. Scale bar: 10 μm for all images. GCL: ganglion cell layer; IPL: inner plexiform layer; INL: inner nuclear layer; OPL: outer plexiform layer; ONL: outer nuclear layer.

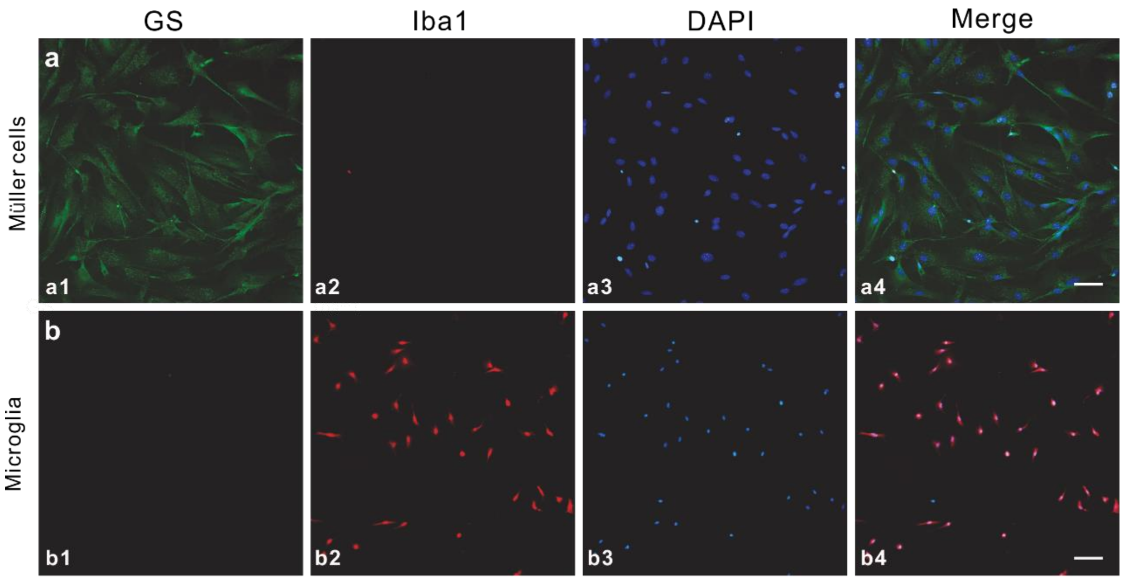

Fig. S5. Identification of purity of cultured retinal Müller cells and microglia. (a, b) Confocal laser microphotographs of cultured retinal Müller cells (a) or microglia (b), stained with the antibodies against GS (green) and Iba-1 (red). Scale bars, 20 µm, for all the images. Nucleus were stained with DAPI. Scale bar: 20 µm for all images. All *in vitro* experiments: n = 3 biological replicates × 3 technical replicates.

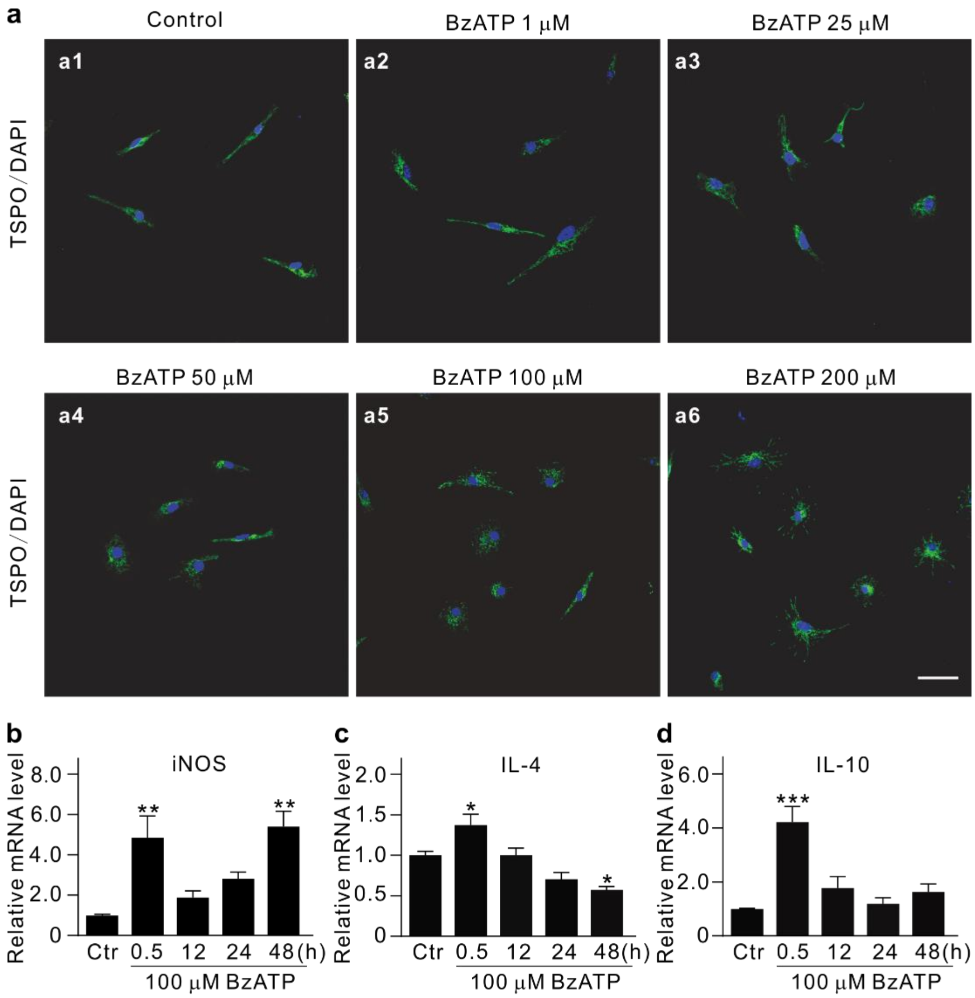

Fig. S6. P2X7R activation-induced functional changes in cultured retinal microglia. **(a)** Representative immunofluorescent images showing the morphological changes and the changes in TSPO expression in primary cultured retinal microglia after the cells were treated with BzATP at different concentrations for 30 min. Nucleus were stained with DAPI. Scale bar: 20 μm for all images. **(b-d)** Bar charts summarizing the changes in mRNA levels of iNOS (b), IL-4 (c), and IL-10 (d) in cultured microglia extracts obtained in Ctr and those with BzATP-treatment for different periods of time. All the data are normalized to Ctr. All *in vitro* experiments: n = 3 biological replicates × 3 technical replicates. *$P < 0.05$, **$P < 0.01$, and ***$P < 0.001$ *vs*. Ctr using ordinary one-way ANOVA with Dunnett's multiple comparisons test (B-D).

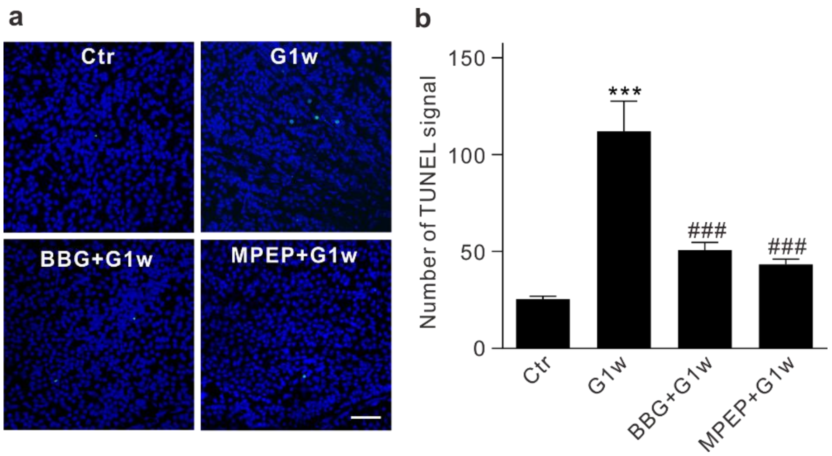

Fig. S7. Müller cell activation and ATP/P2X7R mediated microglia activation contribute to RGC apoptosis in COH retinas. (a) Representative images of TUNEL staining obtained from control (Ctr), G1w, G1w+MPEP, and G1w+BBG retinas. MPEP (20 μM, 2 μL) or BBG (10 μM, 2 μL) was intravitreally injected 1d before the COH operation. All images were taken from whole-flat mounted retinas in the regions at angle 0°. **(b)** Bar charts summarizing the changes in number of TUNEL-positive RGCs under different conditions. n = 6 for each group. *** $P < 0.001$ vs. control (Ctr); $^{###}$ $P < 0.001$ vs. G1w.

Table S1. DNA primers used in qPCR amplification reactions

**Primers sequence**		
β-Actin	Forward	5'-GGCTGTATTCCCCTCCATCG-3'
	Reverse	5'-CCAGTTGGTAACAATGCCATGT-3'
TNF-α	Forward	5'-CCTGTAGCCCACGTCGTAG-3'
	Reverse	5'-GGGAGTAGACAAGGTACAACCC-3'
IL-6	Forward	5'-CTGCAAGAGACTTCCATCCAG-3'
	Reverse	5'-AGTGGTATAGACAGGTCTGTTGG-3'
iNOS	Forward	5'-ACATCGACCCGTCCACAGTAT-3'
	Reverse	5'-CAGAGGGGTAGGCTTGTCTC-3'
IL-4	Forward	5'-ATCATCGGCATTTTGAACGAGG-3'
	Reverse	5'-TGCAGCTCCATGAGAACACTA-3'
IL-10	Forward	5'-AGCCTTATCGGAAATGATCCAGT-3'
	Reverse	5'-GGCCTTGTAGACACCTTGGT-3'
GDNF	Forward	5'-ATGGGATTCGGGCCACTTGG-3'
	Reverse	5'-TCAGATACATCCACACCGTTTAG-3'
LIF	Forward	5'-AATGCCACCTGTGCCATACG-3'
	Reverse	5'-CAACTTGGTCTTCTCTGTCCCG-3'
NGF	Forward	5'-GGCGTACAGGCAGAACCGTA-3'
	Reverse	5'-CAGCCTCTTCTTGTAGCCTTC-3'
CCL2	Forward	5'-GCAGGTCCCTGTCATGCTT-3'
	Reverse	5'-CTAGTTCACTGTCACACTGG-3'
CX3CL1	Forward	5'-TTCACGTTCGGTCTGGTGGG-3'
	Reverse	5'-GGTTCCTAGTGGAGCTAGGG-3'
VCAM	Forward	5'-GGATACCAGCTCCCAAAATCC-3'
	Reverse	5'-CACTTTGGATTTCTGTGCCTC-3'
ICAM	Forward	5'-CCAATTCACACTGAATGCCAG-3'
	Reverse	5'-GGCTTGTCCCTTGAGTTTTATG-3'
